# Caregiver Nutritional Health Outcomes of the Simple Suppers Study: Results from a 10 Week, Two-Group Quasi-Experimental Family Meals Intervention

**DOI:** 10.3390/nu14020250

**Published:** 2022-01-07

**Authors:** Laura C. Hopkins, Christopher Holloman, Alison Webster, Allison N. Labyk, Christine Penicka, Leah May, Amy Sharn, Shivani Gupta, Heather Schier, Julie Kennel, Carolyn Gunther

**Affiliations:** 1 Department of Public Health and Prevention Sciences, Baldwin Wallace (B.W.) University, Berea, OH 44017, USA; lhopkins@bw.edu; 2 Xometry, Gaithersburg, MD 20879, USA; christop.holloman@sbcglobal.net; 3 Food Directions, Shady Side, MD 20764, USA; awebster1124@gmail.com; 4 Cerascreen GmbH, Stadt, 20359 Hamburg, Germany; allison.labyk@gmail.com; 5 Albany Medical Group Pediatric, Albany Medical College, Albany, NY 12208, USA; cmp246@cornell.edu; 6 Wexner Medical Center, The Ohio State University (O.S.U.), Columbus, OH 43210, USA; may.640@osu.edu; 7 Center for Healthy Weight and Nutrition, Nationwide Children’s Hospital, Columbus, OH 43205, USA; amy.sharn@nationwidechildrens.org; 8 Gastroenterology, Hepatology, and Nutrition, Nationwide Children’s Hospital, Columbus, OH 43205, USA; shivani.gupta@nationwidechildrens.org; 9 Department of Human Sciences, Human Nutrition Program, The Ohio State University (O.S.U.), Columbus, OH 43210, USA; schier.8@osu.edu (H.S.); kennel.3@osu.edu (J.K.); 10 College of Nursing, Martha S. Pitzer Center for Women, Children, and Youth, The Ohio State University (O.S.U.), Columbus, OH 43210, USA

**Keywords:** family meals, adult obesity, childhood obesity, caregivers, BMI, blood pressure, diet

## Abstract

Individuals from racial minority backgrounds, especially those in low income situations, are at increased risk for obesity. Family meals positively impact child nutritional health; however, there is limited evidence examining the impact on caregivers, particularly racial minority and income-restricted individuals. The objective of this intervention study was to determine the effect of Simple Suppers, a 10 week family meals program, on caregiver diet and nutrition outcomes. Intervention versus waitlist control participants were compared from baseline (T0) to post-intervention (T1). In addition, intervention participants were assessed at a 10 week follow-up time point (T2). This study was a two-group quasi-experimental intervention trial. Lessons (10 total) were delivered on a weekly basis for 90 min. Data were collected from intervention and waitlist control participants at T0 and T1, and intervention participants at T2. After baseline (T0) data collection, families enrolled in the immediate upcoming session of Simple Suppers (intervention group) or waited for 10 weeks (waitlist control group) to begin the program. Participants were caregivers of children ages 4–10 years. This study was conducted in a faith-based community center for underserved families in Columbus, Ohio. Primary outcomes were: diet quality assessed by Healthy Eating Index (HEI) total and component scores, and total energy intake (kcal/day); body mass index (BMI) (kg/m^2^), waist circumference (cm), systolic and diastolic blood pressure (BP) (mmHG); and self-efficacy for having healthy meals and menu planning (both scalar). The impact of the intervention (T0:T1) was assessed using generalized mixed-effects linear regression models. Maintenance of change in study outcomes among intervention participants (T1:T2) was examined with paired *t*-tests. 109 caregivers enrolled in this study. The retention rate at T1 was 90% (i.e., 98 participants). 56 of 68 intervention participants completed T2, resulting in a retention rate of 82%. Almost all (99%) were female, 61% were Black, and 50% were between 31 and 40 years old. In total, 40% had low income and 37% had low or very low food security. At T1, intervention vs. waitlist controls had a lower daily energy intake (*p* = 0.04), but an HEI-2010 component score for fatty acids (adequacy) that was lower indicating a lower dietary intake of fatty acids (*p* = 0.02), and a component score for empty calories (moderation) that was significantly lower indicating a higher intake of empty calorie foods (*p* = 0.03). At T1, intervention vs. waitlist controls also had a lower BMI (*p* < 0.001) and systolic BP (*p* = 0.04), and higher self-efficacy (*p* = 0.03). There were no group differences in other outcomes. At T2, intervention participants maintained the changes in daily energy intake, BMI, systolic BP, and self-efficacy that improved during the intervention period. There was no change (improvement) in the component score for fatty acids; however, the component score for empty calories significantly improved (*p* = 0.02). Engagement in the Simple Suppers program led to improvements in caregivers’ daily caloric intake, weight status, systolic blood pressure, and self-efficacy for family meals. Future research should further explore the dietary and nutritional health benefits of family meals among caregivers at the highest risk for obesity.

## 1. Introduction

Adult obesity is a persisting and pressing nutrition-related public health problem in the U.S., affecting over 30% of the population [1]. Similar to risk for other non-communicable chronic diseases, individuals from racial minority backgrounds (i.e., Black vs. White) are disproportionately affected by obesity [2], and poverty confounds the problem [3]. The health consequences of obesity are devastating and well documented. Those with obesity are at increased risk for multiple diseases including type 2 diabetes, certain types of cancers, heart disease and heart failure, and hypertension and stroke [4]. Furthermore, in the COVID-19 pandemic, emerging data indicate that individuals with overweight or obesity are at increased risk for medical complications and mortality [5].

Rates of obesity in childhood and adulthood are tightly linked in that adults with obesity are more likely to have been diagnosed with overweight or obesity as children [6]. This is fueled in part by modifiable behavioral factors such as diet [4], which are established early in life [7]. Caregivers have a heavy influence on their children’s dietary intake in part through food-related parenting practices such as role modeling and availability [8]. Thus, interventions aimed at preventing the accumulation of excessive weight (fat mass) in childhood, and engage both children and caregivers, via improved dietary patterns have the greatest potential in terms of a having a marked impact on the obesity epidemic. 

Family meals interventions in particular hold promise based on the literature demonstrating a protective effect of family meal frequency on child nutritional health, including weight status [9,10,11,12,13,14], and the potential to impact caregivers’ own diet, health, and well-being [15,16]. The evidence demonstrating that caregivers place value on family meals and desire to engage their children in the food and meal preparation process provides additional rationale for investing in this line of research [17,18]. Unfortunately, there is limited research examining the effect of family meals on caregivers, especially individuals from diverse racial and socioeconomic backgrounds who are at highest risk for obesity. 

The main objective of this study was to determine the effect of Simple Suppers, a 10 week family meals program tailored to racially diverse and underserved families with school-aged children on caregiver diet and nutrition outcomes [19]. We hypothesized that from baseline (T0) to post-intervention (T1), participants in the intervention versus waitlist control group would demonstrate improved dietary quality (Healthy Eating Index (HEI)-2010 total and component scores), energy intake (kcal/day), body mass index (BMI) (kg/m^2^), waist circumference (cm), blood pressure (BP) (mmHg), self-efficacy for having healthy meals (scalar) and menu planning (scalar). We further hypothesized that participants in the intervention group would demonstrate a maintenance of change in study outcomes during the follow-up period (T1:T2).

## 2. Materials and Methods

### 2.1. Study Design

The complete study protocol is published elsewhere [19]. Briefly, this study was a two-group (intervention and waitlist control) quasi-experimental intervention trial conducted over a 12 month period (2015–2016). There were three cohorts, separated by 10 weeks. After baseline data collection, families enrolled in the immediate upcoming 10 week session of Simple Suppers (intervention group) or chose to wait for 10 weeks (waitlist control group) to begin the program. The waitlist controls did not receive any treatment during the 10 week wait period. Trial Registration Number: NCT02923050; Simple Suppers Scale-up (S3); https://clinicaltrials.gov/ct2/show/NCT02923050, accessed on 23 November 2021; retrospectively registered on 4 October 2016; first participant enrolled on January 2015.

### 2.2. Setting

This study occurred at a faith-based community center offering programming and medical, legal, and other services to those in need of such resources [19]. Service area census tracts at the time of this study demonstrated that the center’s surrounding neighborhoods had a median household income of $32,307–$58,490, 10.7–24.9% were under the federal poverty line (FPL), 41.8% were Black, and 58.7% of households were classified as families. This compared to the broader county as follows: $51,890 household income, 13.2% below the FPL, and 21.2% Black. 

### 2.3. Participants

Research staff and volunteers recruited families through the center (events, newsletter announcements, flyers) [19]. Study eligibility criteria included the following: caregiver of a 4–10 year old child; main food preparer; English as the primary language; resided in the US for ≥1 year. In the case that an eligible caregiver had multiple children between 4 and 10 years old, those children were invited to join this study. Data on child participants are published elsewhere [14].

### 2.4. Data Collection

Baseline (T0) data collection occurred during the two weeks preceding the start of intervention programming [19]. Caregiver consent/permission and child assent were obtained prior to data collection. All data collection appointments (T0, T1, and T2) occurred at participant’s home or the center. Data were collected via questionnaires and direct measure at each study time point. Data collectors went through formal training in all research methods. Families received a $25 grocery store gift card at T0, T1, and T2. The Institutional Review Board at The Ohio State University approved all study materials and procedures. 

### 2.5. Intervention

Intervention mapping was utilized in the development of the Simple Suppers [20] and detailed elsewhere [19]. Briefly, based on the evidence at the time linking family meals with child diet and health [21,22,23,24], the program objectives for the Simple Suppers intervention were to: increase the frequency of family meals prepared in the home and improve child diet. The Social Cognitive Theory was the overarching theoretical framework [25].

Simple Suppers included ten lessons lasting up to 90 min each delivered weekly over the dinner hour at the center [19]. The first 45 min included separate interactive group lessons for adults and children. Caregivers engaged in group discussions and goal setting activities; children were involved in hands-on food preparation activities. During the last 45 min of programming, caregivers and their child(ren) came together and engaged in assembling and having a meal together. Each caregiver lesson topic focused on establishing healthy family mealtime routines: making family mealtime fun; planning family meals on a budget; time-saving strategies for family meals; connecting with your child through family meals; planning well-balanced family meals; re-think your drink; making healthy cooking tasty and easy; serving and eating healthy portions; eating healthy when away from home; and planning fun and healthy snacks.

### 2.6. Outcome Measures

Personal (race, sex, and age) and household (income and food security [26]) characteristics were assessed at T0 using a demographics form. Low income was categorically defined as participation in one or more of the federal food assistance programs (SNAP, WIC, NSLP).

Dietary intake was estimated conducting three non-consecutive (two weekdays, one weekend day) 24 h dietary recalls using the USDA’s 5-step multi-pass dietary recall method [27]. The first dietary recall was conducted during the in-person data collection visit, and the remaining two were conducted via telephone within two weeks of the first recall. Data were entered using the Nutrition Data System for Research, Version 2015 [28]. Fruit (servings/day), vegetables (servings/day), sugar-sweetened beverages (servings/day), and total energy (kcal/day) were calculated averaging across recalls at each time point. Health Eating Index (HEI)-2010 total (100 maximum) and component (5 or 10 maximum) scores were calculated to assess diet quality [29]. A higher HEI-2010 score, total or component (adequacy or moderation), indicates greater conformance with the 2010–2015 Dietary Guidelines for American [30]. Daily calories (kcalories) were also measured.

Anthropometrics (BMI and waist circumference) and Biometrics (BP). A Hopkins Road Rod Portable Stadiometer, Caledonia, USA was used to measure height and a BalanceForm High Accuracy Digital Scale was used for weight [31]. Height and weight were measured twice and averaged, and BMI (kg/m^2^) was calculated [31]. Waist circumference (cm) was measured twice with a tape measure at the uppermost lateral border of the hip crest (ilium) and values averaged [31]. Blood pressure (mmHg) was measured three times via automated blood pressure monitors (Panasonic EW3109W, Osaka, Japan) and values averaged [31].

Self-Efficacy and Menu Planning. Caregiver self-efficacy for having healthy family mealtime routines was assessed with a 12-item questionnaire [32]. The stem statement is: “I can…”, and questions are situated on a 10-point scale (0 = Not confident at all (0 pt), 10 = Very Confident (10 pt)). A sum score was calculated (0–120). Good internal consistency was demonstrated (α = 0.84). Caregiver menu planning was assessed with a 9-item questionnaire, which asks respondents to rate statements regarding menu planning, meal decision-making, and grocery shopping [33]. Statements are situated on a 4-point scale (1 = Never (1 pt), 2 = Sometimes (2 pt), 3 = Often (3 pt), 4 = Always (4 pt)). A sum score was calculated (9–36). Good internal consistency was demonstrated (α = 0.54).

### 2.7. Process Measures

Program dose was assessed by collecting weekly attendance (individual level). Families signed in on an attendance log at the beginning of a lesson; caregivers signed their name and the name(s) of the child(ren) from their family attending that lesson. Program fidelity was assessed using a program-specific checklist during each weekly lesson. Caregivers rated their acceptability of the program with a ‘very unsatisfied’, ‘somewhat unsatisfied’, ‘somewhat satisfied’, or ‘very satisfied’ response to the question.

### 2.8. Sample Size and Data Analysis

Sample size was determined by examining the power of the test for comparing increases in the frequency of family meals (days/week) of the intervention and waitlist control group [19]. Change in the frequency of family dinners was used to power the current study based on the evidence at the time of its effect on the outcome of interest, child BMI [10,22,34,35], and there were no previous studies demonstrating a causal effect of family dinners on BMI. Based on these data, and assuming 20% attrition [34], with an expected effect size of 0.7071, there was 80% power to detect a difference in the frequency of family dinners of 3 days per week with 30 families per group for a total sample size of 60 families at α = 0.05. Since the sample size in the previous pilot was small and uncertainty about estimated effect size was large, a conservative estimate of effect size (i.e., the lower bound of a 95% confidence interval) was used for the power calculation.

Statistical analyses were conducted using Stata [36]. During baseline data collection, three women reported on their own admission that they were pregnant. They were excluded from all dietary and biometric analyses. To evaluate intervention impact, data from the three cohorts were pooled and the intervention tested by comparing change (T1–T0) in study outcomes for the caregivers in the intervention versus caregivers in the waitlist control. Generalized mixed-effects linear regression models (GLMs) were used to test for differences in the response variables of interest between groups at T1, controlling for potential confounders including race (coded categorically: (i) Black, (ii) White, and (iii) Other), income (coded binomially: (i) low income and (ii) non-low income), caregiver sex (coded binomially: (i) female and (ii) male), caregiver age (continuous), baseline (T0) values of the response variables, and cohort (random effect). To evaluate maintenance of change in outcomes among intervention participants, data from the three cohorts (intervention group only) were pooled and change in study outcomes at the end of the 10 week follow-up period was compared. Paired *t*-tests were used to evaluate maintenance of change.

With regard to the dietary data, it was not possible collect all three 24 h dietary recalls in many cases. This was primarily due to challenges in contacting participants. If two recalls were conducted, the average of the two recalls were used, and if a single 24 h dietary recall was collected, the single recall was used. Participant completion of 24 h dietary recalls at T0 was: 0 recalls (10%); 1 recall (64%); 2 recalls (14%); 3 recalls (12%). Participant completion of 24 h dietary recalls at T1 was: 0 recalls (45%); 1 recall (47%); 2 recalls (8%); 3 recalls (0%). Participant completion of 24 h dietary recalls at T2 was: 0 recalls (63%); 1 recall (35%); 2 recalls (2%); 3 recalls (0%).

Multiple imputations were used to account for missing data [36] with the exception of the dietary data due to the high day-to-day variability. Imputation models were built with predicting variables cohort, attendance, number of children, and group assignment. Fifty iterations were run for each missing value and convergence was met. Statistical significance was set at *p* < 0.05.

## 3. Results

**Figure 1:**
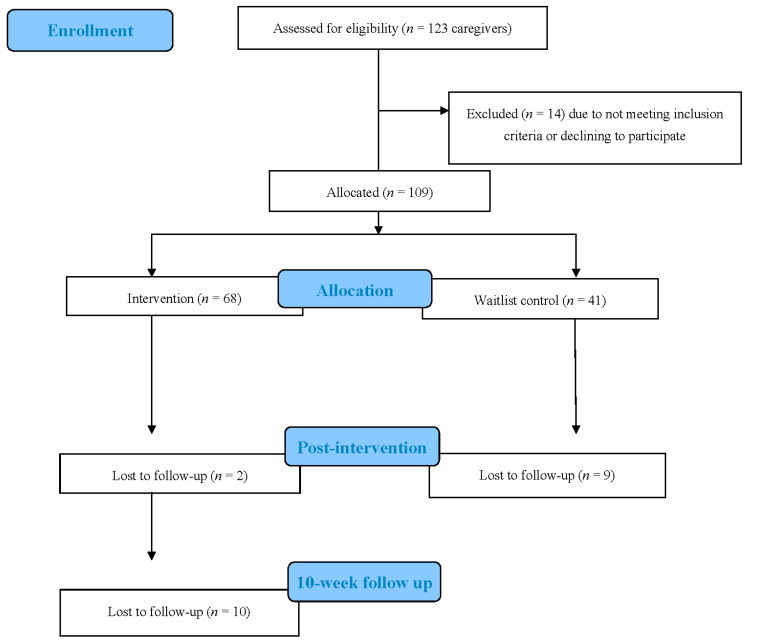
CONSORT Flow Diagram: Caregivers in the 10 Week Quasi-Experimentally Designed Simple Suppers Study.

In total, 109 caregivers enrolled in this study. In total, 98 participants completed T1, resulting in a 90% retention rate (Figure 1). The retention rate at T2 was 82%, with 56 of 68 intervention participants completing assessments. Differences in the CONSORT Flow Diagram numbers between caregivers and children [14] are attributed to the allowance of multiple children from a single household to participate in the intervention. See Table 1 for participant baseline characteristics. No statistical evidence was found indicating a difference in characteristics by group assignment (intervention and waitlist control).

At post-intervention (T1), intervention versus waitlist controls had a significantly lower daily energy intake (*p* = 0.04; CI: −935.94, −15.76), but an HEI-2010 component score for fatty acids that was significantly lower, indicating a lower dietary intake of fatty acids (*p* = 0.02; CI: −2.83, −0.23), and a component score for empty calories that was significantly lower, indicating a higher intake of empty calorie foods (*p* = 0.03; −4.87, −0.22) (Table 2). At T1, intervention versus waitlist controls also had a lower BMI (*p* < 0.001; CI: −2.17, −0.64) and systolic BP (*p* = 0.04; CI: −12.34, −0.21), and higher self-efficacy (*p* = 0.03; CI: 0.70, 11.83) (Table 2). At the 10 week follow up (T2), intervention participants maintained the changes in daily energy intake, BMI, systolic BP, and self-efficacy that improved during the intervention period (T0:T1). There was no change (improvement) in the component score for fatty acids; however, the component score for empty calories significantly improved (*p* = 0.02). See Table 3.

Mean (SD) attendance was 7.16 (2.2) sessions or 72%. In total, 28.6% (*n* = 18) of caregivers attended 4–6 lessons and 8.0% (*n* = 5) attended 1–3 lessons. In total, 95% of the lessons were delivered as intended. Caregivers were engaged 96% of the time. At program completion (T1), 94% of caregivers reported that they were satisfied/very satisfied with the program.

## 4. Discussion

The objective of the current study was to examine if and to what extent participation in a 10 week family meals intervention study impacts caregiver nutritional health outcomes [19]. Caregivers in the intervention versus waitlist control group demonstrated nutritional health outcomes (i.e., lower BMI, lower systolic BP) as well as a lower daily energy intake. Self-efficacy for establishing healthy family mealtime routines also improved in the intervention versus waitlist controls. While caregiver outcomes have been assessed in some of the limited number of published family meals interventions, these studies have been restricted to assessment of dietary intake and personal determinants of behavior [15,16,37]. To the authors’ knowledge, this is the first obesity prevention family meals intervention to expand caregiver outcomes to include direct assessment of anthropometric (BMI) and health (blood pressure) outcomes. Given the persisting obesity epidemic [38], and the need to identify effective intervention strategies, these results are noteworthy.

While there are currently no published results from family meals intervention studies examining caregiver weight status and health outcomes, there are multiple observational studies (all cross-sectional) evaluating the relationship between family meals and caregiver BMI. In general, findings are inconsistent. Results from one study demonstrated an inverse relationship between the frequency of family meals and adult BMI, but only for adults with children in the household [39]. In another study conducted by the same authors, the effect was specific to fathers versus mothers [40]. Somewhat consistent with these findings, data from an Ohio-based survey study of Medicaid participants demonstrated that family meals prepared at home were inversely related to odds of obesity; however, there was no relationship between family meal frequency and obesity risk [41]. On the contrary, other studies have not detected a relationship between family meals and adult BMI. Cross-sectional data from the 2010 Project EAT study demonstrated no association between family meal frequency and parent BMI [42], and the same is true for the 2015–2016 Project EAT study (i.e., no relationship between family meal frequency and parent body size) [43]. There is need for additional research in this area to resolve the inconsistencies—specifically studies with a stronger study design, i.e., longitudinal and intervention in nature, and comprehensive in terms of defining and measuring family meals outcomes (i.e., the frequency of family meals, type and dietary quality of the family meal).

In the current study, intervention impacts were also identified on the psychosocial level. Specifically, caregivers in the intervention versus waitlist control group had greater self-efficacy for having healthy family mealtime routines from pre- to post-intervention. In addition, the increase was retained at the 10 week post-intervention follow up. These results parallel a similar family meals intervention study conducted by Fulkerson et al. that consisted of five parent goal-setting and 10 monthly family meal sessions with an emphasis on nutrition education, meal planning, cooking skill development, and reducing screen time [15]. Intervention parents had significant improvements in self-efficacy for identifying appropriate portion sizes at post-intervention and at a 21 month follow up compared to the control group [15]. Similar to results from the current study, there was not a significant difference in meal planning for intervention parents compared to the control group; however, both groups showed increasing trends over time [15]. Comparably, Fruh et al. conducted a four week family meals intervention at faith-based centers in predominantly African American communities that included a cost-effective cooking demonstration and discussed the benefits of family meals, meal planning assistance, ideas for promoting communication at the table, and strategies to make healthy meals [37]. Following the intervention, 72% of participants reported that they had learned a great deal from the intervention and 75% reported that they learned new information about family meals and their impact on families [37]. Additionally, 93% of participants reported that they had stronger interest in improving family meals because of the program [37]. The Simple Suppers study results corroborate the aforementioned studies and suggest that family meals interventions can be a low-cost, high-impact approach for improving caregiver self-efficacy and knowledge related to family meals.

The observed improvement in BMI (and downstream, BP) among Simple Suppers intervention participants is most likely explained by lower daily energy intake post-intervention—which importantly did occur. In fact, the mean difference in daily energy intake between groups from pre- to post-intervention was −485.85 kcal/day, which, over the 10 week intervention period, closely coincides with the observed 1.4 decrease in BMI. That these changes were maintained during the follow-up period points to the potential of the intervention having lasting or long-term effects, thereby strengthening the overall findings from this study. Future research should explore if and to what extent changes in BMI and BP as a result of participating in this intervention lead to improvements in other health-related biomarkers.

The decrease in daily energy intake in the intervention versus waitlist control participants during the intervention period did not translate into a decline in overall dietary quality represented by the HEI-2010 total component score; however, there were certain other declines in diet quality—specifically the HEI-2010 components scores for fatty acids (i.e., a ratio of polyunsaturated and monounsaturated to saturated fatty acids) [29] and empty calories (i.e., solid fats and added sugars). At the 10 week follow up, when intervention participants demonstrated a maintenance of decreased daily energy intake, there were no improvements in the component score for fatty acids; however, there was importantly a significant improvement in the score for empty calories. These data bear some similarity to the Simple Suppers curriculum itself [19] in that there is a strong emphasis throughout the program on controlling portion sizes and limiting ‘empty calories’, particularly sugar-sweetened beverages, and point to the need to provide a greater emphasis on healthy food substitutions, particularly ‘healthy fats’.

The overarching theoretical framework of the Simple Suppers program is the Social Cognitive Theory [19,44]. A limitation of this theory is that it lacks explicit incorporation of the social support construct, an important determinant of behavior change according to the Social Network Theory [45]. While social support was not factored in or measured in the current study, it is possible that caregivers in the intervention group versus waitlist control developed social support in the form of emotional support, informational support, and appraisal support [45], leading to the observed positive changes in caregiver outcomes. In fact, in a small sample of exit interviews (*n* = 4; data not shown), caregivers reported their appreciation for the chance to meet other families (e.g., “Umm, I guess for me what I liked was we got to meet some new families and some new people and it was nice to just kind of have that time with parents and their kids umm at the church”). The high attendance data (i.e., more than half of participants attended the majority of lessons) provide further evidence in support of receiving social support in being part of the program. Future research should examine if and to what extent Simple Suppers and other similar interventions that bring caregivers and families together build social support and explain change(s) in health behaviors.

The present study had several strengths. To our knowledge, this was the first study of its kind to assess caregiver anthropometric and biometric data from a family meals intervention in a racially and socioeconomically diverse population. In addition, the Simple Suppers intervention was designed using the intervention mapping protocol, the gold standard method in developing theory- and evidence-based health promotion programs [19]. Further, the intervention program was designed in partnership with members of the community, and was delivered by trained nutrition professionals and peer educators [19]. Limitations to this study include a lack of randomization into study groups therefore introducing the chance of selection bias. However, between-group differences were assessed at baseline and differences accounted for in all statistical analyses. Another limitation was that this study was not powered to detect changes in caregiver BMI, thus a causal relationship cannot be established. In addition, the follow-up rates for dietary recall data collection at T2 were low. Therefore, it is plausible that a non-response bias impacted results (i.e., participants who maintained habits 10 weeks post-intervention may have been more motivated to participate in the follow-up data collection). In addition, in many situations, only one dietary recall was collected, thus the dietary data may ultimately not be an accurate representation of participants’ typical intake.

## 5. Conclusions

Participation in Simple Suppers—designed with specificity to improve caregiver participants diet and health-related outcomes—improved daily caloric intake, weight status, systolic blood pressure, and self-efficacy. Given the ongoing obesity epidemic and the urgency to identify effective and scalable approaches, these results are impactful. Future research should be invested in examining the protective effects of healthy family mealtime routines among caregivers at greatest risk for obesity. Specifically, studies should investigate the mechanisms of action behind these positive health outcomes in caregivers; in addition, the specific program attributes that may lead to benefits in family meals and whether participation in general, or specific lessons/educational materials are leading to these improvements in self-efficacy and health outcomes. Further, the reciprocal role of caregiver and child behavior—how child behavior influences the behavior of the caregiver—should be assessed.

## Figures and Tables

**Table 1:** Baseline (T0) Demographics of 98 Caregivers (total and by group assignment) ^a^.

Characteristics	Total (*n* = 98)	Intervention (*n* = 66)	Waitlist Control (*n* = 32)	*p*-Value
Demographics				
Sex (female) ^b^, *n* (%)	*n* = 96	*n* = 65	*n* = 31	0.49
Female	95 (98.96)	64 (98.46)	31 (100.00)	
Male	1 (1.04)	1 (1.54)	0 (0.00)	
Race ^c^, *n* (%)	*n* = 96	*n* = 65	*n* = 31	0.07
Black	59 (61.46)	39 (60.0)	20 (64.52)	
White	28 (29.17)	17 (26.15)	11 (35.48)	
Other ^d^	9 (9.38)	9 (13.85)	0 (0.00)	
Age (years) ^c^, *n* (%)	*n* = 91	*n* = 62	*n* = 29	0.36
18–30	9 (9.89)	7 (11.29)	2 (6.90)	
31–40	46 (50.55)	27 (43.55)	19 (65.52)	
41+	36 (39.56)	28 (45.16)	8 (27.59)	
Household Income Status ^b,e^, *n* (%)	*n* = 91	*n* = 62	*n* = 29	0.81
Low Income	36 (39.56)	24 (38.71)	12 (41.38)	
Non-Low Income	55 (60.44)	38 (61.29)	17 (58.62)	
Home Food Security ^c,f^, *n* (%)	*n* = 94	*n* = 63	*n* = 31	0.10
High/Marginal Food Security	59 (62.77)	35 (55.56)	24 (77.42)	
Low Food Security	19 (20.21)	16 (25.40)	3 (9.68)	
Very Low Food Security	16 (17.02)	12 (19.05)	4 (12.90)	
Anthropometrics and Biometrics ^g^				
BMI (kg/m^2^) ^h^, mean (SE)	*n* = 86	*n* = 58	*n* = 28	0.35
	32.62 (1.13)	31.88 (1.28)	34.16 (2.25)	
Weight Status ^c^, *n* (%)	*n* = 87	*n* = 58	*n* = 28	0.77
Underweight (BMI < 18.5)	2 (2.33)	2 (3.45)	0 (0.00)	
Normal Weight (BMI 18.5–24.9)	22 (25.58)	13 (22.41)	9 (32.14)	
Overweight (BMI 25–29.9)	17 (19.77)	12 (20.69)	5 (17.86)	
Obese (BMI ≥30)	45 (52.33)	31 (53.44)	14 (50.00)	
Waist Circumference (cm) ^h,i^, mean (SE)	*n* = 88	*n* = 59	*n* = 29	0.62
	102.84 (2.23)	102.06 (2.61)	104.43 (4.24)	
Blood Pressure (mmHg) ^h^, mean (SE)	*n* = 90	*n* = 61	*n* = 29	
Systolic Blood Pressure	128.33 (1.72)	127.90 (2.08)	129.22 (3.10)	0.72
Diastolic Blood Pressure	81.94 (1.28)	80.95 (1.41)	84.02 (2.63)	0.26

^a^ Sample sizes are provided for each outcome variable since participant response rate varied. ^b^ Chi-square. ^c^ ANOVA. ^d^ Alaska Native/American Indian, Asian, Native Hawaiian/Pacific Islander, or Mixed Race. ^e^ Low income was defined as participation in one or more of the federal food assistance programs (SNAP, WIC, NSLP). ^f^ USDA 6-item Short Form Home Food Security Questionnaire. A score of 0–1 = High/marginal food security, 2–4 = Low food security; 5–6 = Very low food security. ^g^ *n* = 3 participants excluded from anthropometric and biometric analyses due to pregnancy status. ^h^ *t* test. ^i^ Analysis includes female participants only due to waist circumference recommendations varying based on gender.

**Table 2:** By-Group Impacts of the Simple Suppers Program: Difference at Post-Intervention (T1) Controlling for Baseline (T0) Values.

Outcomes	Intervention			Waitlist Control			β	*p*-Value	95% CI
	T0	T1	Δ	T0	T1	Δ			
Dietary Intake ^a,b,c,d^, mean (SE) (*n* = 40)									
HEI ^e^ Total Score ^f^ (100 maximum)	58.68 (1.87)	55.52 (3.05)	−3.16 (3.43)	58.50 (2.71)	61.57 (2.98)	3.07 (3.94)	−6.53	0.17	(−15.75, 2.69)
HEI Component Scores									
Adequacy									
Total fruits (0–5)	2.00 (0.32)	1.80 (0.42)	−0.20 (0.44)	1.82 (0.48)	2.08 (0.67)	0.26 (0.93)	−0.45	0.53	(−1.86, 0.96)
Whole fruits (0–5)	2.5 (0.38)	1.85 (0.43)	−0.70 (0.46)	2.87 (0.67)	2.21 (0.69)	−0.66 (0.99)	−0.38	0.60	(−1.83, 1.05)
Total vegetables (0–5)	3.00 (0.28)	3.44 (0.31)	0.44 (0.40)	2.56 (0.51)	3.10 (0.50)	0.55 (0.55)	0.04	0.93	(−0.94, 1.03)
Greens and beans (0–5)	2.94 (0.39)	2.17 (0.43)	−0.77 (0.56)	0.97 (0.56)	2.52 (0.68)	1.54 (0.89)	−0.39	0.62	(−1.99, 1.19)
Whole grains (0–10)	4.01 (0.75)	2.24 (0.76)	−1.77 (1.00)	5.39 (1.36)	3.37 (1.15)	−2.01 (1.84)	−1.34	0.28	(−3.80, 1.11)
Dairy (0–10)	5.39 (0.56)	5.26 (0.65)	−0.14 (0.83)	4.82 (0.97)	4.43 (1.04)	−0.39 (1.22)	0.74	0.51	(−1.43, 2.89)
Total protein foods (0–5)	4.49 (0.19)	6.76 (0.58)	2.26 (0.63)	4.68 (0.15)	6.37 (0.94)	1.70 (0.93)	0.57	0.57	(−1.41, 2.54)
Seafood and plant proteins (0–5)	2.94 (0.43)	4.72 (0.86)	1.78 (0.96)	2.5 (0.75)	5.27 (1.40)	2.77 (1.27)	−0.75	0.57	(−3.36, 1.85)
Fatty acids (0–10)	7.70 (0.40)	6.81 (0.39)	−0.89 (0.48)	7.76 (0.81)	7.94 (0.56)	0.18 (1.12)	−1.53	0.02	(−2.83, −0.23)
Moderation									
Refined grains (0–10)	7.86 (0.53)	5.21 (0.74)	−2.65 (0.84)	8.79 (0.45)	6.83 (0.99)	−1.96 (1.13)	−1.39	0.25	(−3.79, 1.00)
Sodium (0–10)	7.38 (0.52)	7.30 (0.60)	−0.08 (0.66)	6.94 (1.21)	5.50 (1.37)	−1.44 (1.21)	1.85	0.08	(−0.23, 3.95)
Empty calories (0–20)	8.40 (0.60)	7.66 (0.77)	−0.74 (1.01)	9.41 (0.53)	9.43 (0.44)	0.02 (0.73)	−2.54	0.03	(−4.87, −0.22)
Total Energy (kcal/day)	2031.79 (108.38)	1885.56 (138.77)	−446.23 (167.54)	1820.91 (184.16)	2014.68 (224.83)	193.78 (197.18)	−485.85	0.04	(−935.94, −15.76)
Anthropometrics and Biometrics ^c,d^									
BMI, mean (SE) (kg/m^2^) (*n* = 94)	31.66 (1.30)	30.85 (1.29)	−0.81 (0.24)	34.39 (2.10)	34.74 (2.15)	0.35 (0.31)	−1.40	<0.001	(−2.17, −0.64)
Waist Circumference, mean (SE) (cm) (*n* = 78) ^g^	99.69 (2.83)	99.47 (2.95)	−0.22 (0.88)	104.85 (4.06)	104.11 (4.25)	−0.73 (0.86)	0.08	0.95	(−2.28, 2.44)
Systolic Blood Pressure, mean (SE) (mmHg) (*n* = 94)	127.69 (2.07)	125.14 (2.26)	−2.55 (2.24)	129.41 (3.05)	130.84 (3.41)	1.43 (2.83)	−6.28	0.04	(−12.34, −0.21)
Diastolic Blood Pressure, mean (SE) (mmHg) (*n* = 94)	80.88 (1.42)	80.15 (1.91)	−0.73 (1.83)	83.90 (2.58)	83.05 (2.84)	−0.85 (2.39)	−1.36	0.64	(−7.15, 4.42)
Personal Determinants^c^, mean (SE) (*n* = 97)									
Self-Efficacy ^h^	94.70 (1.82)	103.61 (2.06)	8.91 (2.15)	96.45 (3.20)	97.26 (2.73)	0.81 (2.89)	6.27	0.03	(0.70, 11.83)
Menu Planning ^i^	24.39 (0.44)	26.36 (0.60)	1.97 (0.58)	24.77 (0.63)	24.60 (0.86)	−0.17 (0.76)	1.73	0.06	(−0.09, 3.55)

^a^ Daily intake averaged across non-consecutive 24 h dietary recalls collected using the USDA five-step multi-pass method. ^b^ Participant completion of 24 h dietary recall at T0: 0 recalls (10%); 1 recall (64%); 2 recalls (14%); 3 recalls (12%). Participant completion of 24 h dietary recalls at T1: 0 recalls (45%); 1 recall (47%); 2 recalls (8%); 3 recalls (0%). ^c^ By-group differences determined by mixed-effects linear regression models with the outcome variable as the T1 value of given outcome, the primary predictor of group assignment (waitlist control = 1, intervention = 2), controlling for household income (low income = 0, non-low income = 1), caregiver race (Other = 1, Black = 2, White = 3), sex (Male = 1, Female = 2), age (18–30 years old = 1, 31–40 years old = 2, 41+ years old = 3), and baseline value of outcome variable, and cohort included as a random effect. ^d^ *n* = 3 pregnant women excluded from analyses. ^e^ HEI = Healthy Eating Index. ^f^ A higher HEI-2010 score, total or component, indicates greater conformance with the 2010–2015 Dietary Guidelines for Americans. ^g^ *n* = 16 outliers excluded from analyses. ^h^ Self-Efficacy for having Healthy Family Meals, 0–120 scale with higher score representing increased self-efficacy. ^i^ Menu Planning, 9–36 scale with higher score representing increased menu planning.

**Table 3:** Within-Intervention Group Differences in Study Outcomes at Baseline (T0), Post-Intervention (T1), and the 10 Week Follow Up (T2).

Dietary Intake ^a,b,c^, Mean (SE)							
Outcomes	T0 (*n* = 54)	Δ T0 to T1 (*n* = 30)	*p*-Value ^i^	Δ T1 to T2 (*n* = 20)	*p*-Value ^i^	Δ T0 to T2 (*n* = 22)	*p*-Value ^i^
HEI ^d^ Total Score ^e^ (100 maximum)	56.11 (1.61)	−3.81 (3.23)	0.25	2.74 (4.41)	0.55	−0.20 (3.11)	0.95
HEI Component Scores							
Adequacy	1.66 (0.24)	−0.46 (0.45)	0.32	0.66 (0.62)	0.30	0.41 (0.47)	0.39
Total fruits (0–5)	2.16 (0.29)	−0.89 (0.45)	0.06	0.28 (0.67)	0.68	0.57 (0.90)	0.54
Whole fruits (0–5)	3.15 (0.22)	0.38 (0.38)	0.32	0.55 (0.54)	0.33	0.70 (0.45)	0.87
Total vegetables (0–5)	2.69 (0.31)	−0.88 (0.54)	0.11	1.10 (0.80)	0.18	−0.10 (0.66)	0.89
Greens and beans (0–5)	4.06 (0.58)	−1.87 (0.94)	0.06	1.35 (1.34)	0.33	−0.26 (1.34)	0.85
Whole grains (0–10)	5.26 (0.43)	0.02 (0.80)	0.98	−0.65 (0.84)	0.45	−1.11 (1.13)	0.34
Dairy (0–10)	4.32 (0.18)	2.22 (0.59)	<0.001	−1.67 (0.70)	0.03	0.56 (0.61)	0.37
Total protein foods (0–5)	2.22 (0.32)	1.66 (0.93)	0.08	−2.02 (0.88)	0.03	0.09 (0.60)	0.88
Seafood and plant proteins (0–5)	7.26 (0.31)	−0.77 (0.46)	0.11	0.98 (0.61)	0.12	0.34 (0.76)	0.66
Fatty acids (0–10)	1.66 (0.24)	−0.46 (0.45)	0.32	0.66 (0.62)	0.30	0.41 (0.47)	0.39
Moderation							
Refined grains (0–10)	7.14 (0.47)	−2.85 (0.80)	<0.01	0.59 (1.46)	0.69	−2.08 (1.32)	0.13
Sodium (0–10)	7.14 (0.42)	0.04 (0.63)	0.95	−0.39 (0.74)	0.60	0.10 (0.82)	0.90
Empty calories (0–20)	8.57 (0.41)	−0.69 (0.95)	0.47	2.40 (0.92)	0.02	1.21 (1.05)	0.26
Total Energy (kcal/day)	1878.31 (97.93)	−445.65 (156.29)	<0.01	−83.13 (235.33)	0.73	−289.36 (142.30)	0.05
**Anthropometrics and Biometrics, mean (SE)**							
**Outcomes**	**T0**	**Δ T0 to T1**	***p*-value ^h^**	**Δ T1 to T2**	***p*-value ^h^**	**Δ T0 to T2**	***p*-value ^h^**
BMI (kg/m^2^) (*n* = 63)	31.66 (1.30)	−0.81 (0.24)	<0.01	−0.04 (0.40)	0.82	−0.85 (0.45)	0.07
Waist Circumference ^f^ (cm) (*n* = 48)	99.69 (2.83)	−0.22 (0.88)	0.80	−0.43 (1.55)	0.79	−0.65 (1.45)	0.66
Systolic Blood Pressure (mmHg) (*n* = 63)	127.70 (2.07)	−2.55 (2.24)	0.26	2.18 (3.10)	0.49	−0.37 (3.20)	0.91
Diastolic Blood Pressure (mmHg) (*n* = 63)	80.88 (1.42)	−0.73 (1.83)	0.69	−1.85 (3.00)	0.55	−2.57 (3.02)	0.40
**Personal Determinants, mean (SE) (*n* = 66)**							
**Outcomes**	**T0**	**Δ T0 to T1**	***p*-value ^h^**	**Δ T1 to T2**	***p*-value ^h^**	**Δ T0 to T2**	***p*-value ^h^**
Self-Efficacy ^g^	94.70 (1.82)	8.91 (2.15)	<0.001	0.31 (1.93)	0.87	9.22 (2.26)	<0.001
Menu Planning ^h^	24.39 (0.44)	1.97 (0.58)	0.001	0.33 (0.47)	0.49	2.31 (0.58)	<0.001

^a^ Daily intake averaged across non-consecutive 24 h dietary recalls collected using the USDA five-step multi-pass method. ^b^ Participant completion of 24 h dietary recalls at T0: 0 recalls (12%); 1 recall (62%); 2 recalls (16%); 3 recalls (10%). Participant completion of 24 h dietary recalls at T1: 0 recalls (49%); 1 recall (40%); 2 recalls (11%); 3 recalls (0%). Participant completion of 24 h dietary recalls at T2: 0 recalls (63%); 1 recall (35%); 2 recalls (2%); 3 recalls (0%). ^c^ *n* = 3 pregnant women excluded from analyses. ^d^ HEI = Healthy Eating Index. ^e^ A higher HEI-2010 score, total or component, indicates greater conformance with the 2010–2015 Dietary Guidelines for Americans. ^f^ *n* = 16 outliers excluded from analyses. ^g^ 0–120 scale with higher score representing increased self-efficacy for having healthy family meals. ^h^ 9–36 scale with higher score representing an increased frequency of menu planning behaviors. ^i^ Within-group differences determined by paired *t*-test.

## Data Availability

The datasets used and/or analyzed during the current study are available from the corresponding author on reasonable request.
